# Alcohol Consumption Trends in Self-poisoning Suicide Attempts During COVID-19: A Seven-Year Retrospective Study

**DOI:** 10.1192/j.eurpsy.2026.11977

**Published:** 2026-07-31

**Authors:** A. Zemaitis, M. Čistov, L. Černius, R. Badaras

**Affiliations:** 1 Faculty of Medicine, Vilnius University, Clinic of Psychiatry, Institute of Clinical Medicine; 2 Republican Vilnius University Hospital; 3 Vilnius University, Faculty of Medicine; 4 Faculty of Medicine, Vilnius University, Clinic of Anaesthesiology and Intensive Care, Institute of Clinical Medicine, Vilnius, Lithuania

## Abstract

**Introduction:**

Alcohol abuse, a known risk factor for suicidality, remains a major global concern. The COVID-19 pandemic significantly affected mental health and substance use. Social restrictions during and after the pandemic may have contributed to suicidality, warranting closer investigation.

**Objectives:**

This study aimed to examine alcohol consumption patterns across different phases of the COVID-19 pandemic, alongside an analysis of indirect alcohol exposure biomarkers and sociodemographic characteristics in patients who attempted suicide by self-poisoning.

**Methods:**

This retrospective study ranged from December 2017 to December 2024 and took place at the Republican Vilnius University Hospital. Phases of the pandemic were defined by the first case of COVID-19 in Lithuania and the end of the state of emergency. The analysis was conducted on 941 hospitalized adult patients with suicide attempts by self-poisoning. Serum ethanol (g/l), collected at the time of hospital admission, indirect alcohol biomarkers (AST, ALT, GGT, MCV), and sociodemographic variables (age, sex, locations by population density) were evaluated.

**Results:**

The Data sample comprised 662 women and 279 men, 368 from low- and 573 from high-density populations. The median age was 42 years. Alcohol intoxication was observed in 407 cases, with a median ethanol concentration of 0.18g/l. Ethanol concentration showed moderate correlation with ALT, GGT, and MCV, but weak correlation with AST (all p<0.001). Men were more likely to be intoxicated (p<0.001), and intoxication was more prevalent in smaller densities (p=0.004). Indirect alcohol biomarkers were significantly associated with intoxication frequency, where abnormal results were linked to intoxication (all p<0.001). The AST/ALT ratio (Median 1.27) showed a significant relationship with gender and intoxication status, where a ratio <1 was associated with higher ethanol concentrations and a ratio >2 was higher in women and non-intoxicated cases.

**Conclusions:**

Alcohol intoxication rates did not differ across pandemic phases. Alcohol exposure by associated biomarkers strongly differentiates intoxicated vs non-intoxicated self-poisoning patients, with gender and location influencing intoxication prevalence.

**Disclosure of Interest:**

None Declared

